# Unveiling the therapeutic potential of airpotato yam rhizome against colorectal cancer: a network pharmacology approach

**DOI:** 10.3389/fonc.2024.1414766

**Published:** 2024-08-02

**Authors:** Yiwen Xie, Sumei Xu, Zhiyun Chen, Caiping Song, Wenxi Yan

**Affiliations:** ^1^ Department of General Practice, The First Affiliated Hospital of Zhejiang Chinese Medical University (Zhejiang Provincial Hospital of Chinese Medicine), Hangzhou, Zhejiang, China; ^2^ Key Laboratory of Integrative Chinese and Western Medicine for the Diagnosis and Treatment of Circulatory Diseases of Zhejiang Province, The First Affiliated Hospital of Zhejiang Chinese Medical University (Zhejiang Provincial Hospital of Chinese Medicine), Hangzhou, Zhejiang, China; ^3^ Department of Rehabilitation, The First Affiliated Hospital of Zhejiang Chinese Medical University (Zhejiang Provincial Hospital of Chinese Medicine), Hangzhou, Zhejiang, China; ^4^ Department of Clinical Laboratory, The First Affiliated Hospital of Zhejiang Chinese Medical University (Zhejiang Provincial Hospital of Chinese Medicine), Hangzhou, Zhejiang, China

**Keywords:** airpotato yam rhizome, colorectal cancer, network pharmacology, molecular docking, PI3K/AKT signaling pathway

## Abstract

**Objective:**

The objective of this investigation was to elucidate the key active compounds and molecular mechanisms underlying the therapeutic potential of airpotato yam rhizome (AYR) in colorectal cancer (CRC) treatment.

**Methods:**

By utilizing network pharmacology and molecular docking, key targets and signaling pathways of AYR against CRC were predicted and subsequently validated in cellular and mouse xenograft models.

**Results:**

This study initially predicted that quercetin was the primary compound in AYR that might have potential efficacy against CRC and that EGFR and AKT1 could be the main targets of AYR, with the EGF/EGFR-induced PI3K/AKT signaling pathway potentially playing a crucial role in the anti-CRC effects of AYR. Molecular docking analysis further indicated a strong binding affinity between quercetin and EGFR, primarily through hydrogen bonds. Additionally, the AYR-derived drug-containing serum was found to inhibit the PI3K/AKT signaling pathway, as demonstrated by decreased levels of p-PI3K, p-AKT, and BCL2, which ultimately led to enhanced apoptosis of HCT116 and HT29 cells. The potential antitumor effects of AYR were investigated in nude mouse xenograft models of human HCT116 and HT29 cells, in which AYR was found to induce tumor cell apoptosis and inhibit tumor formation.

**Conclusion:**

AYR may promote CRC cell apoptosis by suppressing the PI3K/AKT signaling pathway, which provides a basis for further research on the safe and effective use of AYR for the treatment of CRC.

## Introduction

Colorectal cancer (CRC) is widely recognized as the third most prevalent form of cancer and the second leading cause of cancer-related mortality on a global scale (1–3). Projections indicate that the incidence of CRC is expected to increase by 60%, exceeding 2.2 million new cases and 1.1 million fatalities by the year 2030 (4). Owing to the absence of distinctive early clinical manifestations of CRC and the absence of an ideal screening protocol, a majority of patients are confirmed to be in intermediate to advanced stages, which leads to a dismal 5-year overall survival rate of only 12% for advanced patients (5). Consequently, CRC has emerged as a significant public health concern worldwide, posing a serious threat to human wellbeing.

Currently, chemotherapy remains the primary treatment modality for advanced CRC (3, 6, 7). However, conventional cytotoxic chemotherapy agents not only target tumor cells but also induce significant toxicity and adverse effects in healthy tissues, thereby compromising the overall quality of life of patients (8, 9). Therefore, alternative therapies with reduced toxicity are vital for enhancing the clinical efficacy and minimizing treatment-related complications. Currently, adjuvant therapies, such as targeted therapy and immunotherapy, have become increasingly prevalent in clinical practice. The emergence of immunotherapy that involves the immune checkpoint PD-1, monoclonal antibodies, and TCR-T cells has enhanced the precision of cancer immunotherapy, and this approach holds significant potential (10, 11).

Additionally, traditional Chinese medicine (TCM) plays an important and pivotal complementary role in the management of cancer (12–14). It is crucial to maximize the potential benefits of TCM in the treatment of CRC (15, 16). Airpotato yam rhizome (AYR), which is the tuber of *Dioscorea bulbifera* L., has a rich history of medicinal use and is documented in *Materia Medica of Southern Yunnan*. AYR has various effects, such as antitumor, anti-inflammatory, antibacterial activities, antiviral activities, antidiabetic activities, analgesic activities, and antioxidant activities (17). Modern pharmacological research has demonstrated significant therapeutic advantages of AYR in combating various types of cancer, including lung cancer (18) and cervical cancer (19). A recent study revealed that AYR induced apoptosis in HCT116 human colorectal carcinoma cells by inhibiting the ERK 1/2 pathway and activating the JNK signaling pathway (20). However, the clinical utility of AYR has been limited because of its hepatotoxic effects and unclear mechanism of action (21–23). A previous study revealed that diosbulbin B might serve as the primary hepatotoxic chemical compound in AYR owing to its ability to inhibit antioxidant enzymes in liver mitochondria and the activity of drug-metabolizing enzymes (24). In addition, AYR may cause side effects such as nephrotoxicity and toxicity to the gastrointestinal system and thyroid glands (17). Hence, it is imperative to investigate the primary bioactive compounds and pharmacological molecular mechanisms of AYR that target CRC.

In the modern era, the advancement of big data has propelled network pharmacology, which incorporates systems biology, multitarget pharmacology, computational biology, and network analysis, to the forefront of TCM research (25). Furthermore, network pharmacology offers insights into the intricate interplay between drugs and disease-associated targets from a network standpoint, facilitating the exploration of the correlation between diseases and TCMs (26–28).

**Figure 1:**
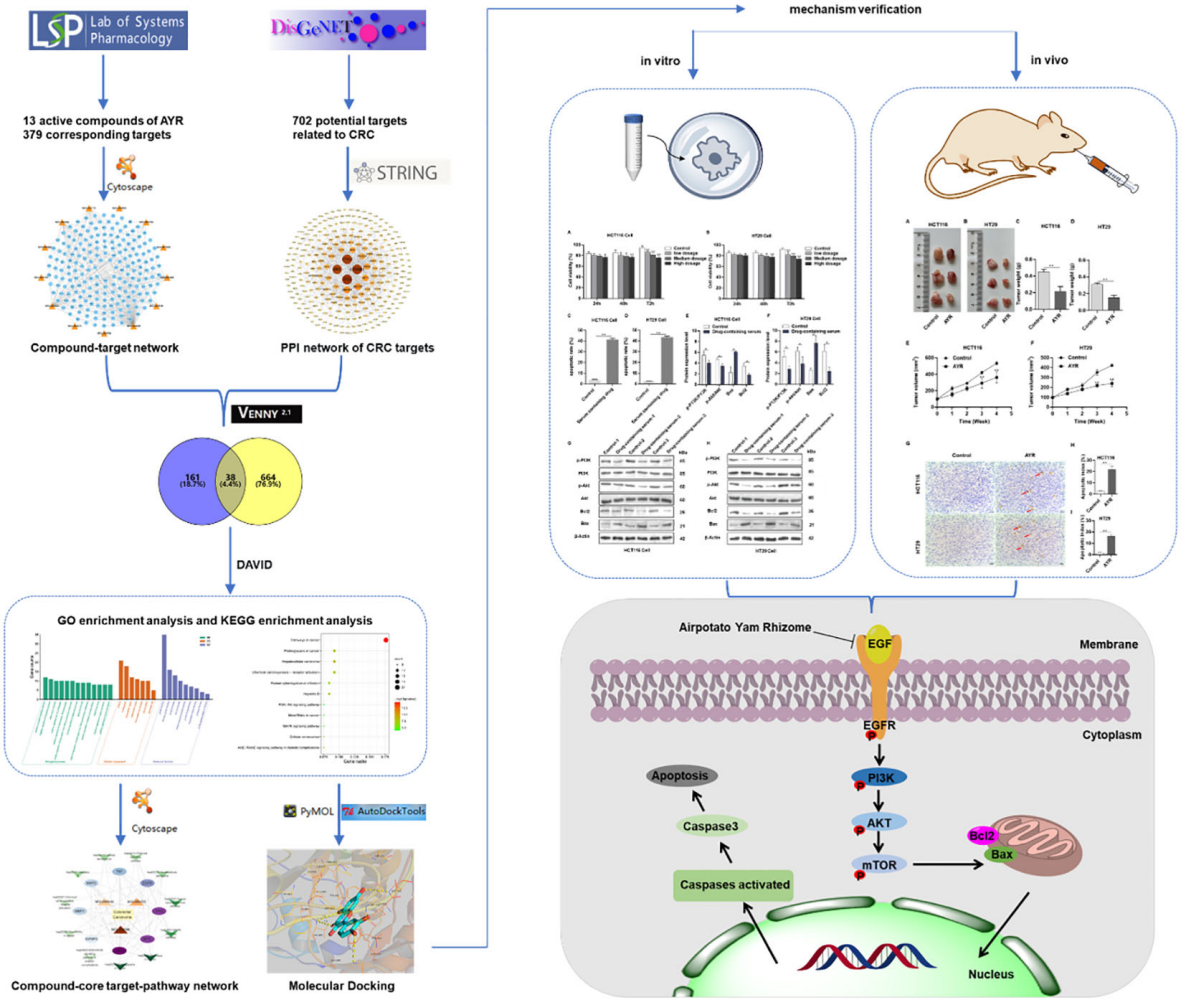
Flowchart of the study of anti-CRC effects of AYR based on network pharmacology prediction and mechanism verification.

In this study, we predicted the major active compounds and signaling pathways of AYR for anti-CRC treatment based on network pharmacology and molecular docking. Subsequently, the putative molecular mechanisms of AYR against CRC were validated through *in vivo* and *in vitro* experiments. **Figure 1** shows a schematic representation of the workflow.

## Materials and methods

### Screening of the active chemical compounds and corresponding targets of AYR

All the AYR compounds were sourced from publicly available databases, specifically the Traditional Chinese Medicines for Systems Pharmacology Database and Analysis Platform (TCMSP; http://tcmspw.com/tcmsp.php), which was utilized for the selection of compounds and the prediction of their targets according to the absorption, distribution, metabolism, and excretion (ADME) criteria. Bioactive compounds associated with AYR were selected based on their oral bioavailability (OB) and drug likeness (DL). Compounds that satisfied the ADME criteria (i.e., the OB threshold ≥30% and the DL threshold ≥0.18) and had related targets were exclusively included in the study for further analysis. Using the UniProt database (https://www.uniprot.org/), which is renowned for providing valuable protein information, protein target names were converted into their respective gene symbols. Subsequently, Cytoscape 3.9.1 was used to construct a compound-target network diagram.

### CRC-related target prediction and screening

By utilizing “Colorectal cancer” as the primary search term, a multitude of targets were retrieved from the DisGeNET database (https://www.disgenet.org/search), which serves as a dedicated resource for disease-related targets and has the largest publicly available collection of genes and variants associated with human diseases. Cytoscape 3.9.1 and STRING (https://string-db.org) were subsequently used to construct a protein−protein interaction (PPI) network.

### Mapping the relationships of shared targets of AYR and CRC

Using an online Venn diagram tool (https://bioinfogp.cnb.csic.es/tools/venny/), a Venn diagram representing the interaction between AYR and CRC was created to identify their shared targets. Subsequently, the shared targets were analyzed using the STRING database with a focus on the species *Homo sapiens* and a confidence score >0.900.

### GO and KEGG analyses

The shared targets were then analyzed using R-Studio and submitted to the DAVID website (https://david.ncifcrf.gov/tools.jsp) for Gene Ontology (GO) enrichment analysis and Kyoto Encyclopedia of Genes and Genomes (KEGG) pathway enrichment analysis. After applying corrections for statistical significance (*p* ≤ 0.05) and controlling for the false discovery rate (FDR < 0.05), the top pathways enriched with the targets were identified for visualization using bioinformatics tools (http://www.bioinformatics.com.cn/). GO serves as a database for functional cluster analysis and interpretation of protein and gene functions within an organism, encompassing cell component (CC), molecular function (MF), and biological process (BP) categories. KEGG provides a comprehensive repository of genomic, chemical, and systematic functional information.

### Network construction analyses

The network topology structure was further analyzed using the NetworkAnalyzer tool within the Cytoscape 3.9.1 software. The major active compounds with a degree of connectivity greater than or equal to 2 were selected based on the betweenness centrality of the node, which reflects the degree of connectivity of compounds, target proteins, and signaling pathways. A diagram illustrating the compound–core target–pathway network was then constructed.

### Target-compound molecular docking

Molecular docking, a crucial technique in network pharmacology that involves the combination of known proteins with small compounds, was utilized. The three-dimensional crystal structure of the EGF-R protein was obtained from the Protein Data Bank (PDB; http://www.pdbus.org/). The removal of small-molecule structures and water molecules within the target protein structure was carried out by utilizing the AutoDock Tools 1.5.6 software. The molecular formula of quercetin was retrieved from PubChem (https://pubchem.ncbi.nlm.nih.gov/). Subsequently, molecular docking between the key target protein and the active compound was conducted with AutoDock Vina. The minimum binding energy and pertinent data structure were then determined through analysis of van der Waals, electrostatic, and hydrogen bond interactions between the ligand and the receptor. Visualization of the binding model was accomplished using the PyMOL 2.3.0 software.

### Extraction and quantitation of AYR

A total of 1,000 g, 500 g, and 250 g of AYR powder were sequentially added to 3–5 L round-bottom flasks, followed by the addition of water at a 1:5 material-to-liquid ratio. The resulting mixtures underwent soaking and agitation and were then transferred to electric heating casings for heating and refluxing over a period of 4 h. Subsequently, the extract solutions were concentrated under reduced pressure using a rotary evaporator. The resulting high-, medium-, and low-concentration AYR extract powders were obtained by lyophilization. Quantification of quercetin in the extracts was performed via LC−MS/MS analysis, with a range of quercetin standard solutions (10.0, 50.0, 100.0, 200.0, 500.0, and 1,000.0 ng/mL) chosen as the linear ranges for quantification. The standard equation derived from the LC-MS/MS detection method was *Y* = 605.12*X−*10,013 (*r* = 0.9992). The findings demonstrated a strong linear correlation within the specified concentration range. According to the standard curve, the quercetin contents in high-, medium-, and low-concentration AYR extracts were 8,263.86 ng/mL, 5,824.60 ng/mL, and 3,418.29 ng/mL, respectively, as illustrated in **Supplementary Figures S1** and **S2**.

### Preparation and analysis of an AYR drug-containing serum

High, medium, and low doses (40 g/kg, 20 g/kg, and 10 g/kg) of the drug were obtained by utilizing varying concentrations of the extracts dissolved in ultrapure water. These designated high, medium, and low doses were then administered to male Sprague–Dawley rats via gavage for a period of 1 week. Blood samples were collected from the abdominal aorta of the rats 1–2 h after the final gavage (29–31), followed by centrifugation at a low temperature to isolate the upper serum. Quercetin concentrations in the serum were determined through LC−MS/MS analysis (**Supplementary Figure S1**).

### Cell culture

The human colon cancer cell lines HCT116 and HT29 were obtained from the National Collection of Authenticated Cell Cultures (NCACC, Shanghai, China). HCT116 and HT29 cells were cultured in McCoy’s 5A medium and RPMI-1640 medium, respectively, supplemented with 10% fetal bovine serum, 100 units/mL penicillin, and 100 µg/mL streptomycin at 37°C with 5% CO_2_.

### Cell viability assay

HCT116 and HT29 cells were seeded in 96-well plates at a density of 5×10^3^ cells/well and exposed to the drug-containing serum (0, 2.5%, 5%, or 7.5%) for 24, 48, or 72 h. Subsequently, the viability of HCT116 and HT29 cells was assessed with a CCK-8 kit (Dojindo, Japan) at a wavelength of 450 nm.

### Flow cytometry analysis of apoptosis

HCT116 and HT29 cells were treated with the AYR-containing serum (7.5%) for 48 h, with normal mouse serum serving as a control. The cells were subsequently harvested, washed twice with cold phosphate-buffered saline (PBS; Sangon Biotech, Shanghai, China), and resuspended in 500 µL of 1× binding buffer containing 5 µL of annexin V-FITC and 5 µL of a PI staining solution (Sigma, USA). The cells were then incubated in the dark at room temperature for 5–15 min for flow cytometry analysis.

### Western blotting assay

HCT116 and HT29 cells were treated with the AYR drug-containing serum (7.5%) for 48 h, with normal mouse serum used as a control (*n* = 3). Total protein was extracted from the cells using a total protein extraction kit (including a protease inhibitor cocktail; Thermo Pierce, USA) and quantified with a BCA kit (Thermo Pierce, USA). Then, the proteins were separated using sodium dodecyl sulfate−polyacrylamide gel electrophoresis (SDS−PAGE) and transferred onto polyvinylidene fluoride (PVDF) membranes (Millipore, USA). Following blocking with Tris-buffered saline containing Tween 20 and 5% bovine serum albumin (BSA; Sangon Biotech, Shanghai, China) for 1 h at room temperature, the membranes were probed with primary antibodies against p-PI3K (1:500, cat. no. ab182651, Abcam), PI3K (1:1,000, cat. no. 4257, CST), p-AKT (1:1,000, cat. no. 4060, CST), AKT (1:1,000, cat. no. 4691, CST), Bax (1:2,000, cat. no. ab182733, Abcam), Bcl2 (1:2,000, cat. no. ab182858, Abcam), and β-actin (1:10,000, cat. no. ab68477, Abcam) at 4°C overnight. Subsequently, the membranes were incubated with secondary antibodies: goat anti-mouse (1:5,000, cat. no. 31160, Thermo Pierce) or goat anti-rabbit (1:5,000, cat. no. 31210, Thermo Pierce) at room temperature for 1 h. Protein bands were detected and visualized using the SuperSignal® West Dura extended duration substrate (Thermo Pierce, USA).

### Animal experiments

Female BALB/C nude mice (4 weeks old, 12–15 g) were procured from Shanghai Slake Laboratory Animal Co., Ltd., and acclimated for 1 week under a 12-h light/dark cycle. The mice were randomly allocated into four groups (*n* = 3) as follows: HCT116/Control (HCT116 tumor formation group administered normal saline intragastrically), HCT116/AYR (HCT116 tumor formation group administered a high dose of AYR intragastrically, 40 g/kg), HT29/Control (HT29 tumor formation group administered normal saline intragastrically), and HT29/AYR (HT29 tumor formation group administered a high dose of AYR intragastrically, 40 g/kg). Tumor xenograft models were established by subcutaneously injecting HCT116 or HT29 tumor cell suspensions (200 μL, 2×10^8^ cells) into the necks of nude mice. The tumor-bearing mice were orally administered the treatments twice daily for 4 weeks. Subsequently, the mice were euthanized, and the subcutaneous transplanted tumors were excised, weighed (g), and subjected to TUNEL staining. The tumor volume was calculated as follows: *V* = *L* (length) × *W* ^2^ (width)/2.

### Statistical analysis

Statistical analysis was performed using the GraphPad Prism 8.0 software, and the data are presented as the mean ± standard deviation (x± s). A Student’s *t*-test was used for comparisons between two groups. One-way ANOVA was used for multiple group comparisons. Statistical significance was denoted as *p < 0.05, while high significance was denoted as **p < 0.01.

## Results

### Compounds and targets of AYR

**Figure 2:**
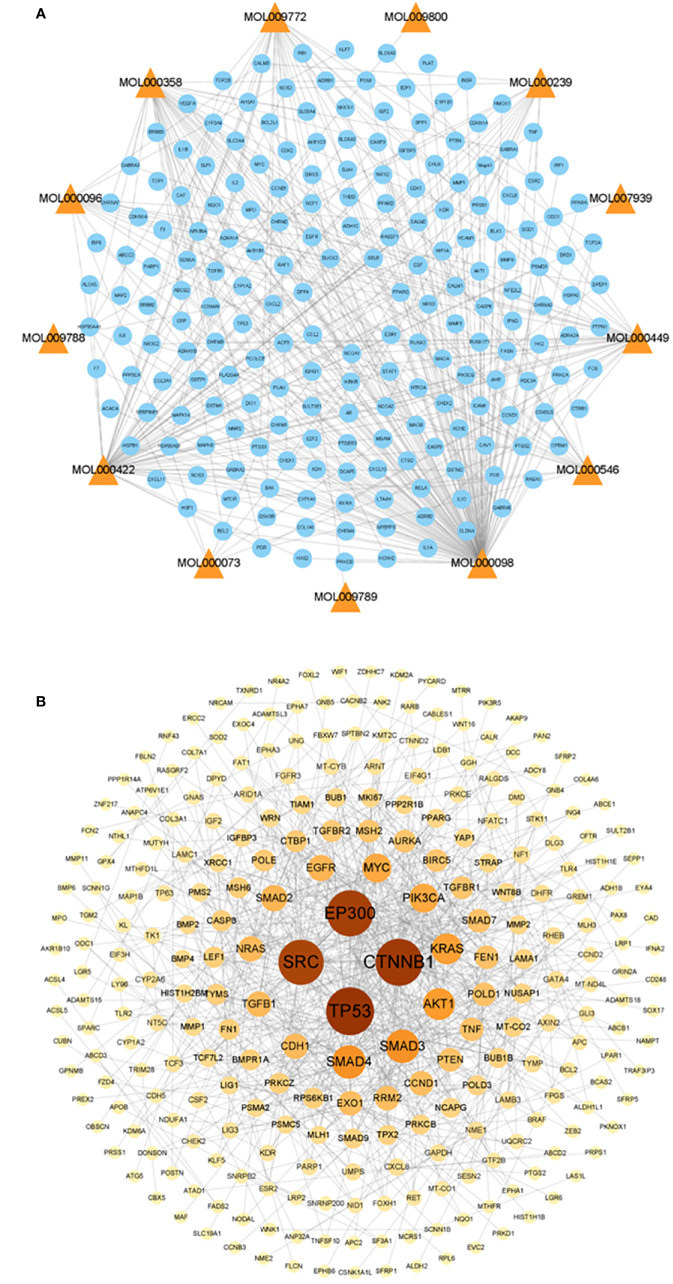
Compound-target network and the PPI network of CRC targets. **(A)** Compound-target network of AYR. The active compounds are shown as orange triangles, and the targets are shown as blue circles. **(B)** PPI network of CRC targets. The nodes are positively sized and colored based on their degree values.

Thirteen active AYR compounds and 379 corresponding targets were identified in the TCMSP database following a drug-likeness (DL) principle ≥ 0.18 and an OB ≥ 30% (**Table 1**). The 13 compounds and their predicted targets are presented in a compound-target network diagram created with Cytoscape 3.9.1 (**Figure 2A**).

**Table 1:** The 13 active compounds of AYR and their chemical properties.

Number	Mol ID	Compound	MW	AlogP	Hdon	Hacc
1	MOL000239	Jaranol	314.31	2.09	2	6
2	MOL000358	Beta-sitosterol	414.79	8.08	1	1
3	MOL000422	Kaempferol	286.25	1.77	4	6
4	MOL000449	Stigmasterol	412.77	7.64	1	1
5	MOL000546	Diosgenin	414.69	4.63	1	3
6	MOL000073	ent-Epicatechin	290.29	1.92	5	6
7	MOL007939	Diosbulbin B	344.39	1.22	0	6
8	MOL000096	(−)-Catechin	290.29	1.92	5	6
9	MOL009772	3,5,3’-Trimethoxyquercetin	374.37	2.05	2	8
10	MOL009788	Diosbulbin A	376.44	0.87	1	7
11	MOL009789	Diosbulbin C	362.41	0.62	2	7
12	MOL000098	Quercetin	302.25	1.5	5	7
13	MOL009800	Kryptogenin	430.69	3.51	2	4

### The CRC target network

A total of 702 potential pathogenic targets associated with CRC were retrieved from the DisGeNET database, and their interaction relationships were visualized in a PPI network (**Figure 2B**). Notably, four targets, TP53 (degree = 40), CTNNB1 (degree = 39), EP300 (degree = 38), and SRC (degree = 37), were identified as playing significant roles in CRC formation.

### PPI network of shared AYR compound targets and CRC targets

**Figure 3:**
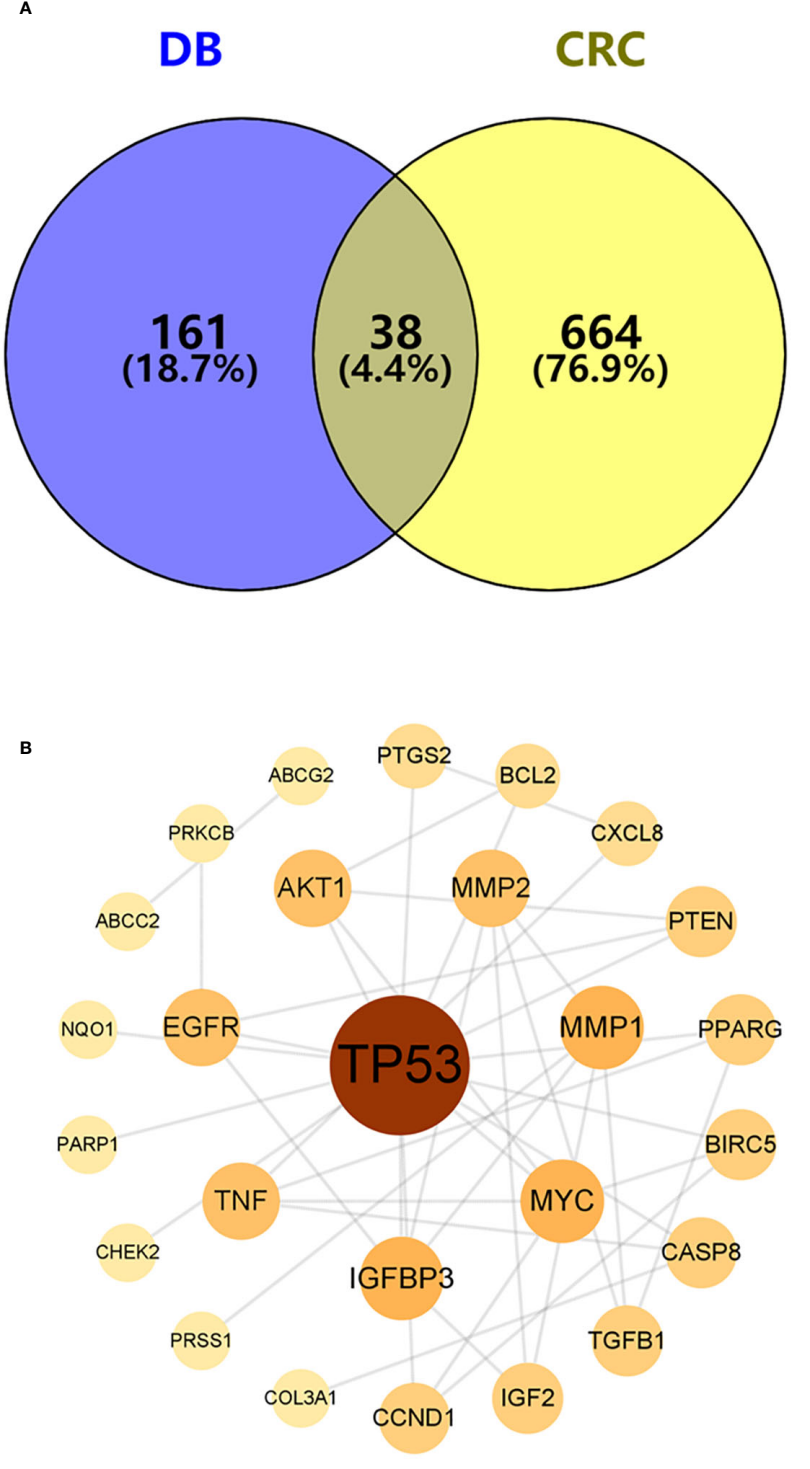
Venn diagram and the PPI network of overlapping targets. **(A)** Venn diagram of the overlapping targets of AYR and CRC. **(B)** PPI network of the overlapping targets. The nodes are positively sized and colored based on degree values.

To identify potential therapeutic targets against CRC, a Venn diagram was constructed using the 379 predicted targets of AYR and 702 CRC-related targets (**Figure 3A**). A total of 38 shared targets were identified and are displayed in a concentric circle based on their degree, with higher degrees indicating a greater anti-CRC importance (**Figure 3B**, **Table 2**). These targets included TP53 (degree = 14), IGFBP3 (degree = 5), MYC (degree = 5), MMP1 (degree = 5), MMP2 (degree = 4), AKT2 (degree = 4), EGFR (degree = 4), and TNF (degree = 4).

**Table 2:** The 38 shared targets.

Target name	Degree	Target name	Degree
TP53	14	PARP1	1
IGFBP3	5	COL3A1	1
MYC	5	NQO1	1
MMP1	5	CHEK2	1
AKT1	4	ABCG2	1
EGFR	4	PRKCB	1
MMP2	4	PRSS1	1
TNF	4	CYP1A2	0
TGFB1	3	GSTM1	0
BIRC5	3	ACACA	0
PPARG	3	KDR	0
PTEN	3	DPEP1	0
CCND1	3	MAP2	0
IGF2	3	RUNX1T1	0
CASP8	3	ODC1	0
CXCL8	2	NR3C2	0
PTGS2	2	CYP1B1	0
BCL2	2	ESR2	0
ABCC2	1	MPO	0

### GO and KEGG enrichment analyses

**Figure 4:**
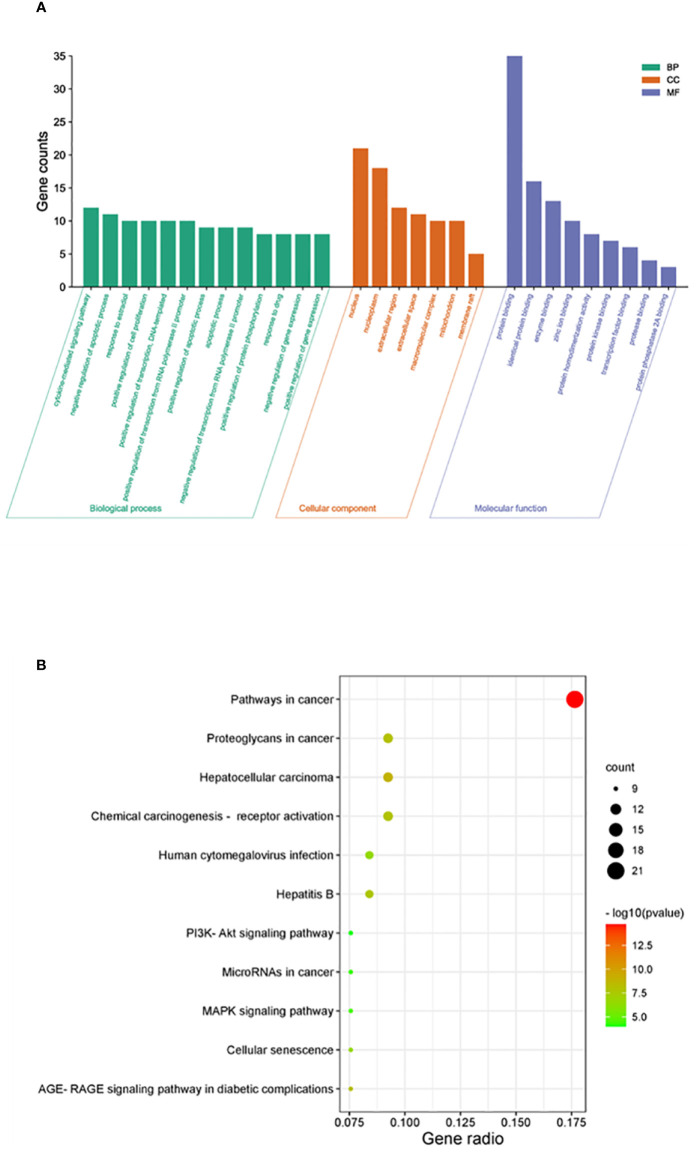
Enrichment analysis of crucial targets. **(A)** GO enrichment analysis of crucial targets, including BPs (green), CCs (orange), and MFs (blue). **(B)** KEGG enrichment analysis of crucial targets. The nodes are positively sized, and the color correlates with the gene ratio involved in the pathways.

DAVID was used for GO and KEGG enrichment analyses of the 38 overlapping targets. Through GO enrichment analysis with a corrected *p*-value of ≤0.05 and an FDR of <0.05, 89 BP, 9 MF, and 7 CC terms were identified. The top 10 GO terms were filtered according to gene counts for visualization (**Figure 4A**). The shared targets were predominantly located in the nucleus (GO:0005634) and primarily interacted through protein binding (GO:0005515). The BPs involving overlapping targets were found to play significant roles in various cellular pathways, including cytokine-mediated signaling (GO:0019221), negative regulation of apoptosis (GO:0043066), response to estradiol (GO:0032355), and apoptosis (GO:0006915) (**Figure 4A**). Through analysis of enriched genes by KEGG, a total of 11 signaling pathways were identified and used to construct a bubble map with a significance level of *p* ≤ 0.05, with pathways in cancer emerging with the highest number of enriched targets (**Figure 4B**).

### Compound–core target–pathway network of AYR against CRC

**Figure 5:**
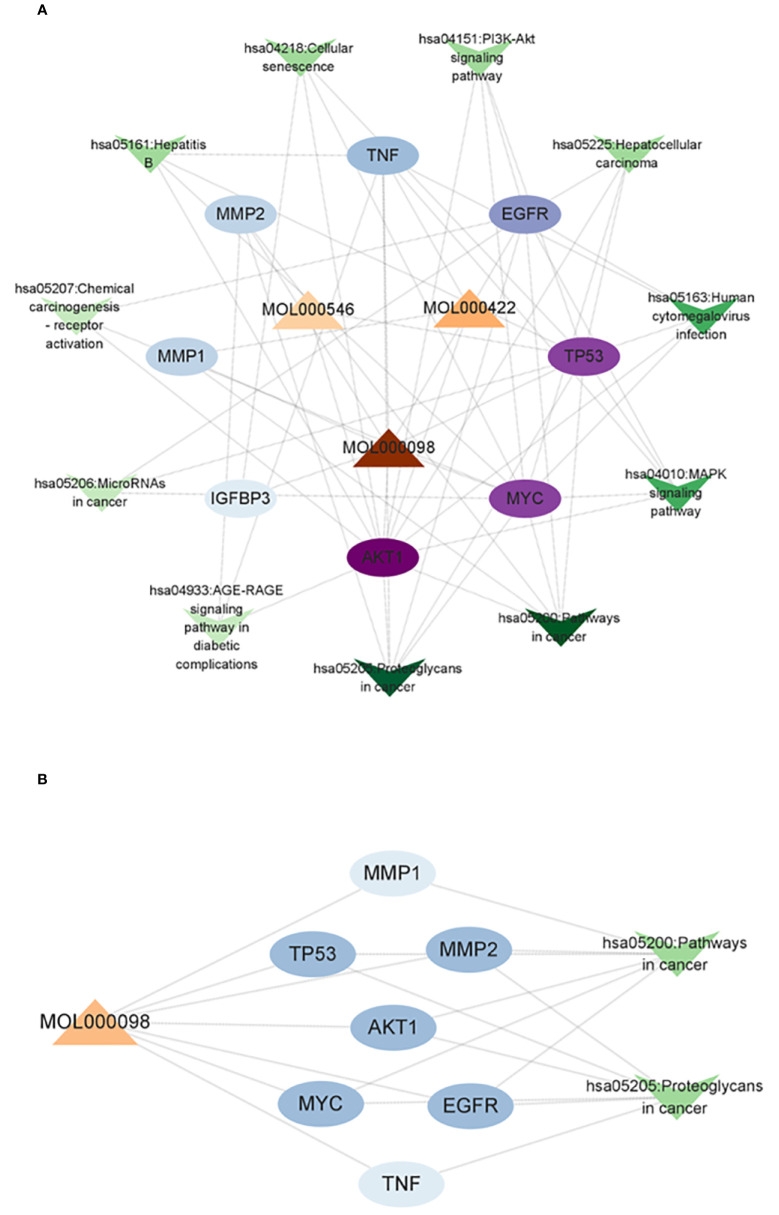
Compound–core target–pathway network of the anti-CRC effect of AYR. **(A)** Compounds are denoted by triangle nodes, core targets are indicated by circular nodes, and signaling pathways are denoted by inverted triangle nodes. The color of the nodes is positively correlated with their degree values. **(B)** Quercetin-related targets enriched in key signaling pathways.

A compound–core target–pathway network was established by analyzing the connectivity of compounds, targets, and signaling pathways with a degree of at least 2. The compounds with a degree of at least 2 were quercetin (degree = 8), kaempferol (degree = 3), and diosgenin (degree = 2) (**Figure 5A**). As one of the crucial anti-CRC compounds in AYR, quercetin was further analyzed by constructing a subnet. This analysis allowed for a more detailed examination of the relationships between quercetin and the first two pathways. The results indicated that the “Pathways in cancer” and “Proteoglycans in cancer” pathways might be critical for the effect of AYR on CRC, with the core targets identified as AKT1, MYC, TP53, and EGFR (**Figure 5B**). Thus, we identified a signaling pathway that includes two crucial molecules, AKT1 and EGFR, as the PI3K/AKT signaling pathway.

### Molecular docking analysis

**Figure 6:**
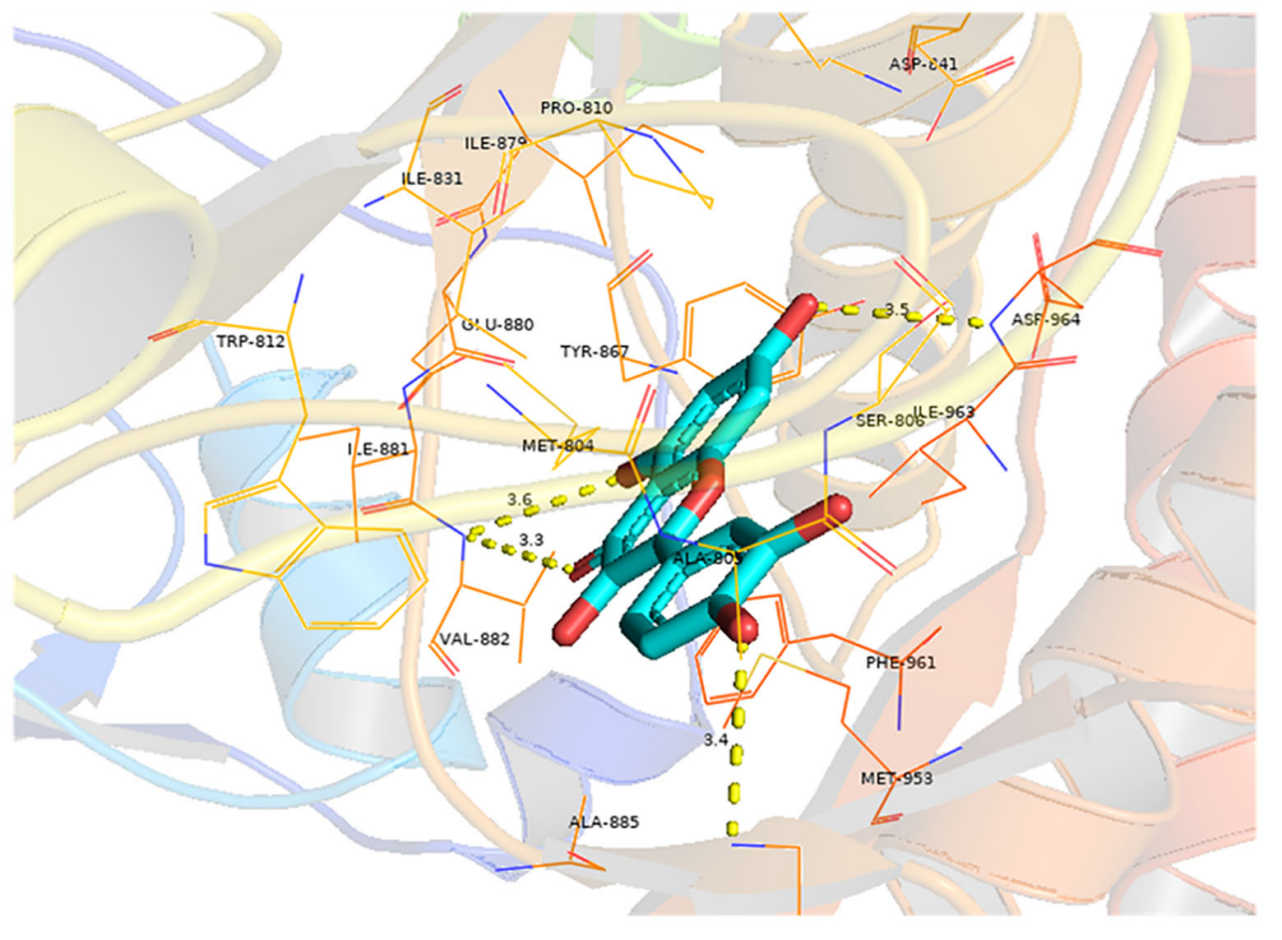
Molecular docking of quercetin with EGFR. The light blue image represents quercetin. The protein target bound to quercetin is shown as a blue rod, and the binding sites are linked by yellow hydrogen bonds. The length of the hydrogen bond is indicated on the side of the bond.

Molecular docking analysis was conducted to investigate the interaction between quercetin and EGFR, with the aim of determining whether quercetin targets EGFR. The results indicated a strong binding affinity (−8.9 kcal/mol) between quercetin and EGFR, primarily through hydrogen bonds (**Figure 6**). Compounds with a higher docking affinity bind more strongly to targets.

### AYR drug-containing serum suppressed the proliferation of CRC cells

**Figure 7:**
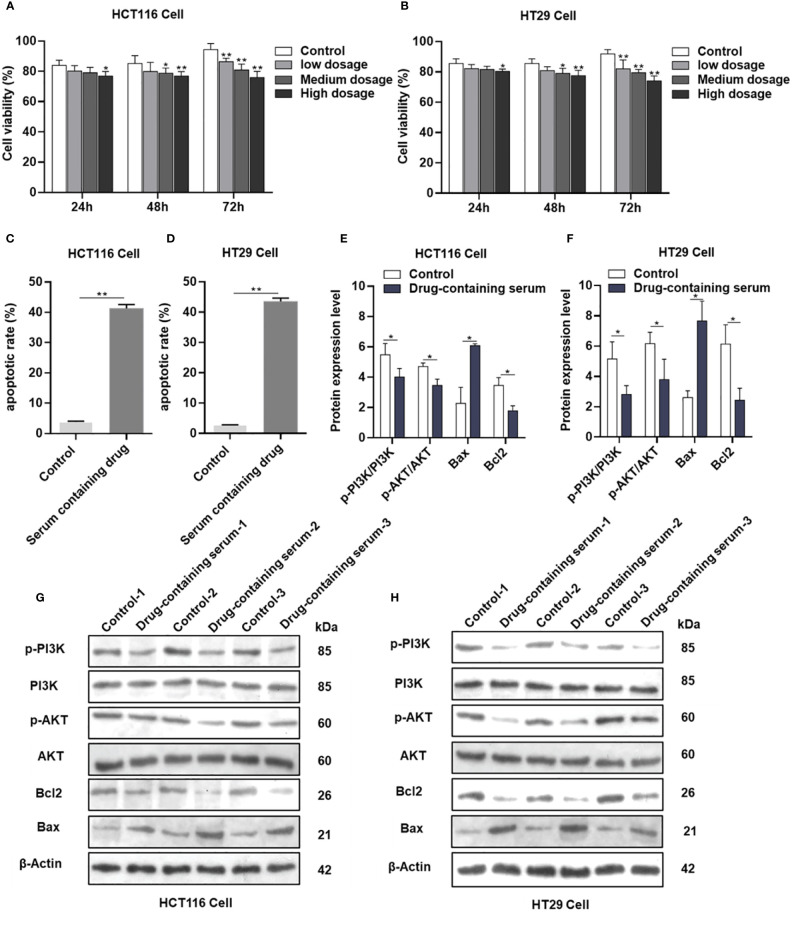
AYR promoted the apoptosis of CRC cells by inhibiting the PI3K/AKT pathway *in vitro*. **(A, B)** Proliferation of HCT116 and HT29 cells after treatment with AYR drug-containing serum for 24, 48, or 72 h. **(C, D)** Percentages of apoptotic CRC cells after treatment with AYR drug-containing serum for 48 h. **(G, H)** Western blots showing the expression of apoptosis-related proteins in HCT116 and HT29 cells. β-Actin was used as an internal reference. **(E, F)** Expression levels of the above proteins are shown in histograms. *p < 0.05, **p < 0.01 vs. control.

The viability of HCT116 and HT29 cells was assessed following treatment with various concentrations (2.5%, 5%, and 7.5%) of drug-containing serum derived from the female nude mice administered AYR for 24, 48, and 72 h using the CCK-8 assay. The inhibitory effects of AYR on the proliferation of HCT116 and HT29 cells were found to be dependent on both the concentration and duration of exposure. Specifically, the high-dose groups exhibited significantly greater inhibition rates (11.78%, 16.29%, and 30.74% against HCT116 cells and 8.53%, 13.11%, and 30.97% against HT29 cells) than did the control groups at 24, 48, and 72 h, respectively (**Figures 7A, B**).

### AYR drug-containing serum induced apoptosis of CRC cells

Compared with those in the control groups, the levels of apoptosis (approximately 41.27% in HCT116 cells and 43.5% in HT29 cells) significantly increased in response to treatment with a high concentration (7.5%) of the AYR-containing serum for 48 h (**Figures 7C, D**). These findings suggested that the AYR drug-containing serum had the potential to induce apoptosis of CRC cells.

### AYR promoted the apoptosis of CRC cells by inhibiting the PI3K/AKT pathway *in vitro*

The previous results indicated that the PI3K/AKT pathway might be crucial for the anti-CRC effect of AYR. Western blot analysis revealed significant decreases in the protein levels of p-PI3K, p-AKT, and Bcl2 and significant increases in the protein levels of BAX compared to those in the control groups (**Figures 7E–H**). These findings suggest that the AYR drug-containing serum may induce apoptosis by blocking the PI3K/AKT pathway in HCT116 and HT29 cells.

### AYR inhibited tumor formation by promoting the apoptosis of CRC cells *in vivo*

**Figure 8:**
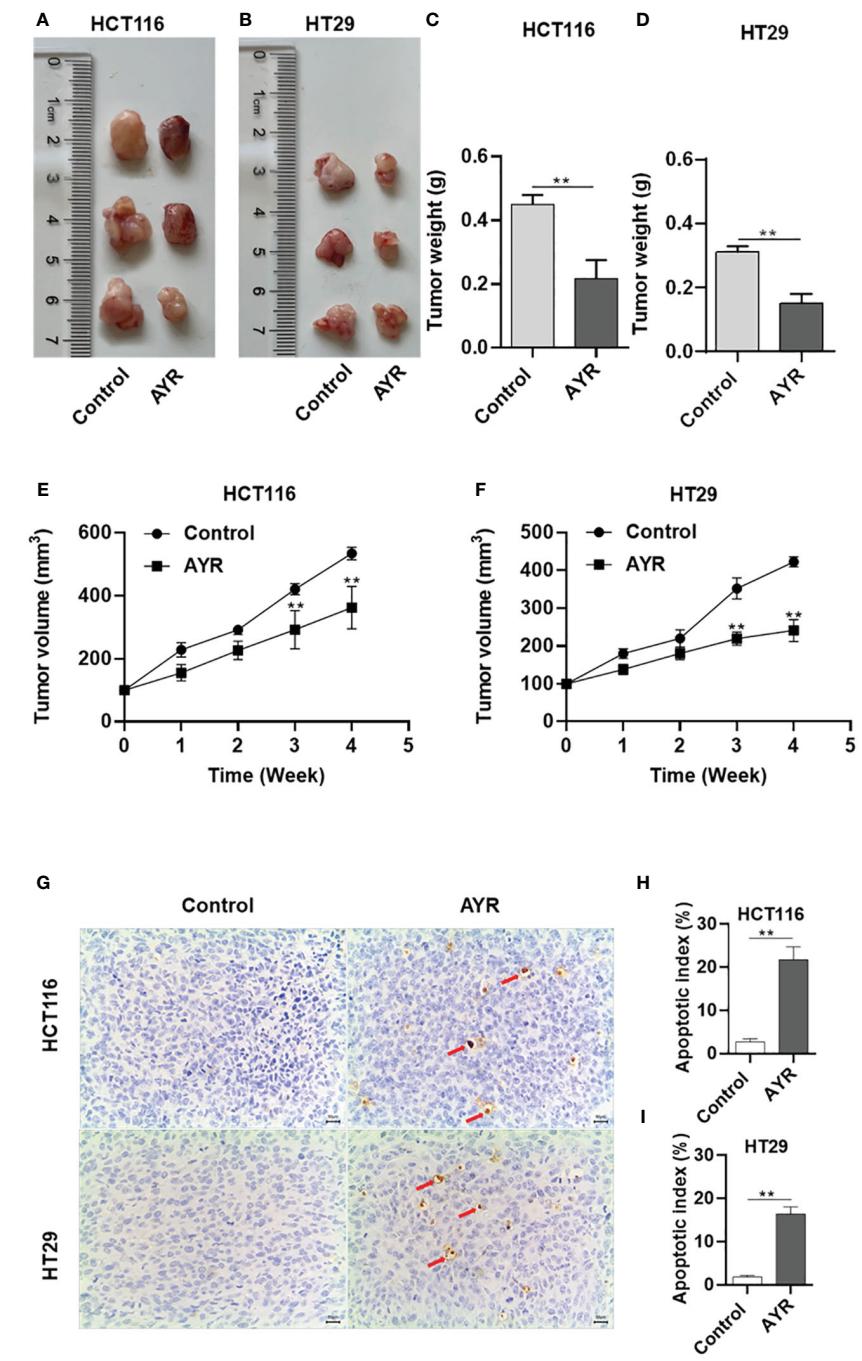
AYR inhibited tumor formation by promoting the apoptosis of CRC cells *in vivo*. **(A, B)** Images of tumors harvested after 4 weeks of treatment with AYR. **(C, D)** Tumor weights in mice after 4 weeks of AYR treatment. **(E, F)** Changes in tumor volumes in tumor-bearing mice after treatment with AYR. **(G)** TUNEL staining of tumor tissues from mice after 4 weeks of AYR treatment (red arrows indicate red-brown apoptotic cells). **(H, I)** Apoptosis indices of tumor tissues from the mice described in **(G)**. **p < 0.01 vs. control.

Nude mouse xenograft models of human HCT116 and HT29 cells were established to evaluate the anti-CRC efficacy of AYR *in vivo*. AYR extract was continuously administered via gavage for a period of 4 weeks. The volumes of the xenograft tumors in the AYR groups were significantly smaller at 3 and 4 weeks, and the tumor weights were significantly smaller after 4 weeks of treatment than those in the respective control groups. These findings indicate that AYR extract can suppress tumor growth in nude mice. Moreover, TUNEL staining of tumor tissues revealed notably greater apoptosis indices in the AYR groups (approximately 16.35%) than in the control groups (approximately 2.75%). Collectively, these results suggest that AYR extract effectively inhibits tumor formation by inducing apoptosis of CRC cells in nude mice (**Figures 8A–I**).

## Discussion

This study predicted the molecular mechanism underlying the AYR effect in the treatment of CRC using network pharmacology. A comprehensive analysis was conducted using data from the TCMSP and DisGeNET databases, which resulted in the identification of 13 active AYR compounds and 379 corresponding drug targets from TCMSP and 702 potential CRC-related pathogenic targets from DisGeNET. A total of 38 shared targets were identified via a Venn diagram. PPI network analysis revealed interactions among the above shared targets, leading to the identification of 38 shared genes that could serve as potential therapeutic targets for CRC, including TP53, IGFBP3, MYC, MMP1, MMP2, AKT2, EGFR, and TNF. Analysis of the 38 shared targets through GO revealed that the interactions between CRC and AYR predominantly occurred within the nucleus and were facilitated by protein interconnections. The primary mechanisms involved in these interactions were found to be cell signal transduction and apoptosis. In addition, KEGG analysis indicated that AYR might exert its effects through “pathways in cancer” signaling pathways. Subsequently, we constructed a network consisting of disease–core target–pathway–compound interactions and postulated that quercetin played a pivotal role in the therapeutic potential of AYR against CRC. Additionally, we investigated two prominent signaling pathways and their associated targets influenced by quercetin. Notably, our findings highlighted the significance of EGF, EGFR, and their downstream pathways in mediating the antitumor effects of AYR. To further elucidate the interaction between quercetin and the core target EGFR, molecular docking analysis was conducted, revealing a strong binding affinity between quercetin and EGFR.

The aforementioned predictions were further corroborated through *in vitro* and *in vivo* experiments. The CCK-8 assay demonstrated that the treatment with the AYR drug-containing serum decreased the viability of HCT116 and HT29 cells. Additionally, the annexin V-FITC and PI assay revealed that the AYR drug-containing serum promoted the apoptosis of HCT116 and HT29 cells. Subsequent Western blot analysis indicated that the AYR drug-containing serum inhibited the PI3K/AKT signaling pathway by reducing p-PI3K, p-AKT, and BCL2 levels, thereby promoting the apoptosis of HCT116 and HT29 cells.

Furthermore, our study validated the inhibitory effects of AYR extract on tumor growth in nude mouse xenograft models of human HCT116 and HT29 cells. Additionally, TUNEL staining demonstrated a greater incidence of apoptosis in the AYR-treated groups than in the control groups.

In summary, this research integrated network pharmacology prediction and experimental verification to elucidate the anti-CRC mechanism of AYR.

Our study has several limitations. It is worth noting that our analysis may have overlooked some bioactive compounds and targets owing to the dynamic nature of public databases. In addition, the mechanism of AYR in the treatment of CRC needs further exploration. Despite these limitations, our findings offer valuable preliminary insights into the potential anti-CRC effects of AYR, indicating its potential as a promising candidate for the treatment of CRC.

Future research will focus on refining the active compounds of AYR and exploring its combinations with other drugs or therapeutic approaches to enhance AYR efficacy and reduce its toxicity in CRC treatment.

## Data availability statement

The datasets presented in this study can be found in online repositories. The names of the repository/repositories and accession number(s) can be found in the article/**Supplementary Material**.

## Ethics statement

The studies involving humans were approved by Ethics Committee of the First Affiliated Hospital of Zhejiang Chinese Medical University. The studies were conducted in accordance with the local legislation and institutional requirements. Written informed consent for participation was not required from the participants or the participants’ legal guardians/next of kin in accordance with the national legislation and institutional requirements. The animal study was approved by Ethics Committee of the First Affiliated Hospital of Zhejiang Chinese Medical University. The study was conducted in accordance with the local legislation and institutional requirements.

## Author contributions

YX: Data curation, Methodology, Writing – original draft, Writing – review & editing, Formal Analysis, Funding acquisition, Software, Visualization. SX: Writing – original draft, Resources. ZC: Supervision, Writing – review & editing. CS: Funding acquisition, Writing – original draft. WY: Writing – original draft, Conceptualization, Funding acquisition, Investigation, Project administration, Validation, Writing – review & editing.
