# Supplementary material for: Unveiling the therapeutic potential of airpotato yam rhizome against colorectal cancer: a network pharmacology approach
Source: Front Oncol. 2024 Aug 2;14:1414766. doi: 10.3389/fonc.2024.1414766 (PMC11327141; doi:10.3389/fonc.2024.1414766)
Supplement: Supplementary file 1 [file Presentation_1.pptx]

## Slide 1
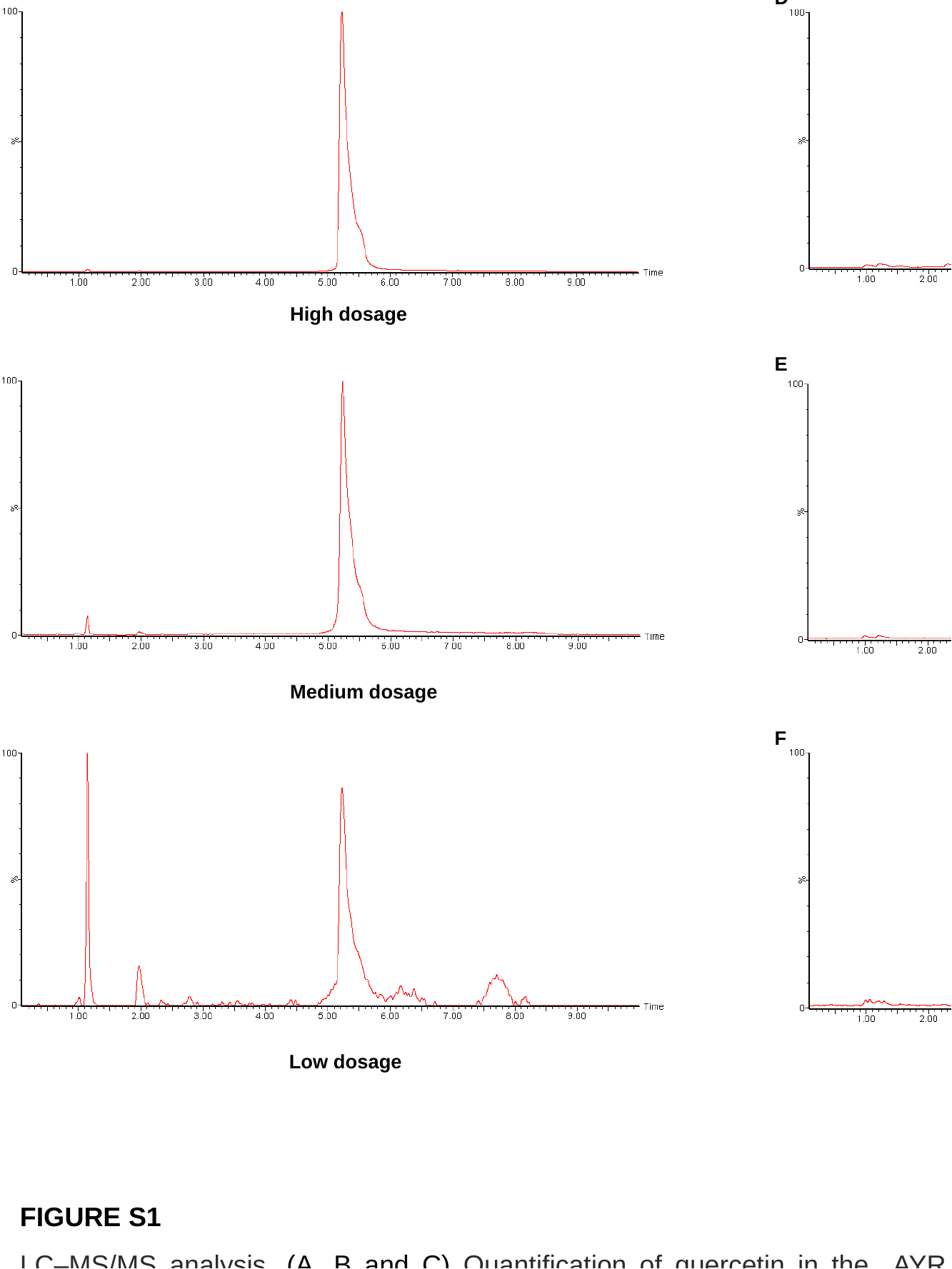

A
D
High dosage
High dosage
B
E
Medium dosage
Medium dosage
C
F
Low dosage
Low dosage
FIGURE S1
LC‒MS/MS analysis. (A, B and C) Quantification of quercetin in the AYR extracts was performed via LC‒MS/MS analysis. (D, E and F) Quercetin concentrations in the AYR drug-containing serum were determined through LC‒MS/MS analysis.

## Slide 2
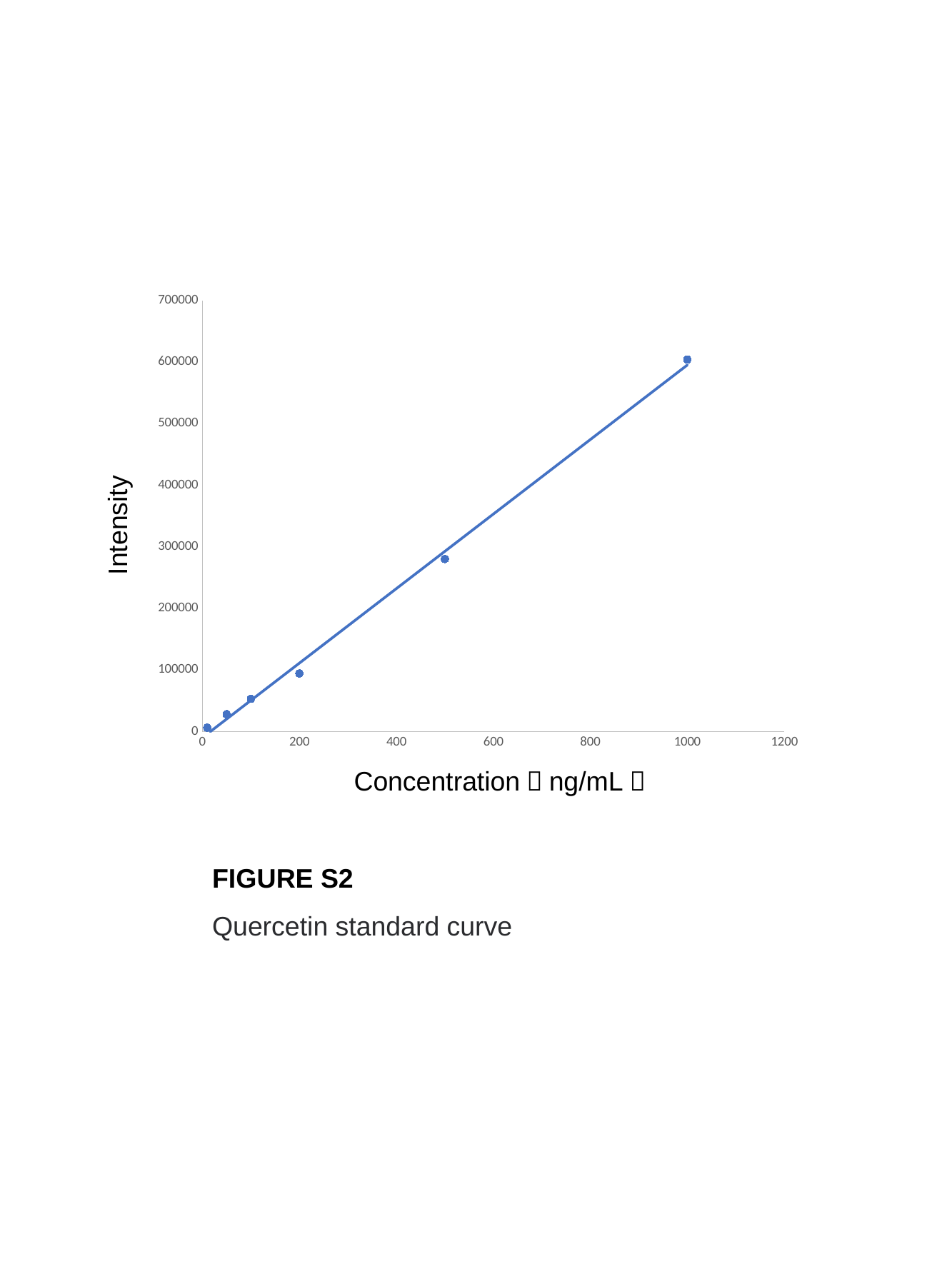

### Chart
| Category | |
|---|---|Intensity
Concentration（ng/mL）
FIGURE S2
Quercetin standard curve
